# Antimicrobial and Antibiofilm Activities of Carvacrol, Amoxicillin and Salicylhydroxamic Acid Alone and in Combination vs. *Helicobacter pylori:* Towards a New Multi-Targeted Therapy

**DOI:** 10.3390/ijms24054455

**Published:** 2023-02-24

**Authors:** Valentina Puca, Gabriele Turacchio, Beatrice Marinacci, Claudiu T. Supuran, Clemente Capasso, Pamela Di Giovanni, Ilaria D’Agostino, Simone Carradori, Rossella Grande

**Affiliations:** 1 Department of Pharmacy, “G. d’Annunzio” University of Chieti-Pescara, 66100 Chieti, Italy; 2 Institute of Endocrinology and Experimental Oncology “Gaetano Salvatore”, National Research Council, 80131 Naples, Italy; 3 Department of Innovative Technologies in Medicine and Dentistry, “G. d’Annunzio” University of Chieti-Pescara, 66100 Chieti, Italy; 4 Neurofarba Department, Section of Pharmaceutical and Nutraceutical Sciences, University of Florence, Sesto Fiorentino, 50019 Florence, Italy; 5 Department of Biology, Agriculture and Food Sciences, National Research Council (CNR), Institute of Biosciences and Bioresources, 80131 Naples, Italy

**Keywords:** *Helicobacter pylori*, carvacrol, amoxicillin, biofilm, carbonic anhydrases, salicylhydroxamic acid, urease

## Abstract

The World Health Organization has indicated *Helicobacter pylori* as a high-priority pathogen whose infections urgently require an update of the antibacterial treatments pipeline. Recently, bacterial ureases and carbonic anhydrases (CAs) were found to represent valuable pharmacological targets to inhibit bacterial growth. Hence, we explored the underexploited possibility of developing a multiple-targeted anti-*H. pylori* therapy by assessing the antimicrobial and antibiofilm activities of a CA inhibitor, carvacrol (CAR), amoxicillin (AMX) and a urease inhibitor (SHA), alone and in combination. Minimal Inhibitory (MIC) and Minimal Bactericidal (MBC) Concentrations of their different combinations were evaluated by checkerboard assay and three different methods were employed to assess their capability to eradicate *H. pylori* biofilm. Through Transmission Electron Microscopy (TEM) analysis, the mechanism of action of the three compounds alone and together was determined. Interestingly, most combinations were found to strongly inhibit *H. pylori* growth, resulting in an additive FIC index for both CAR-AMX and CAR-SHA associations, while an indifferent value was recorded for the AMX-SHA association. Greater antimicrobial and antibiofilm efficacy of the combinations CAR-AMX, SHA-AMX and CAR-SHA against *H. pylori* were found with respect to the same compounds used alone, thereby representing an innovative and promising strategy to counteract *H. pylori* infections.

## 1. Introduction

*Helicobacter pylori* eradication is strongly suggested in patients colonized by this bacterium in order to reduce the incidence of gastric cancer [1,2,3,4]. The eradication of the microorganism is challenging because of its genetic variability, its capacity to form biofilm and its ability to develop resistance against commonly used antimicrobials [4,5,6,7,8]. *H. pylori* is capable of forming biofilm on human gastric mucosa as a strategy for colonization, protecting itself from the attack of antimicrobials and the host immune system [9,10]. Bacterial biofilms are more recalcitrant to the action of antimicrobial agents because of the Extracellular Polymeric Substances (EPS) matrix which slows down the process of reaching target sites [11,12].

According to the Maastricht V Consensus Report, standard triple therapy, which includes a Proton Pump Inhibitor (PPI) plus clarithromycin and amoxicillin (AMX) or metronidazole, continues to be the first-choice therapy. [13]. However, in 2017 the World Health Organization (WHO) included *H. pylori* in the list of pathogens for which new antimicrobials are urgently needed as a result of its increasing resistance to antibiotics, placing it in the high-priority category [14], because of its status as a public health threat [8] and its classification as a Group 1 Carcinogen by the International Agency for Research on Cancer [15]. Based on these concerns, the identification of new strategies or adjuvant compounds to standard antimicrobial therapy is required to counteract *H. pylori* infection. In particular, bacterial proteins/enzymes to be used as novel targets need to be investigated in order to reach this goal. Novel approaches to prevent biofilm formation and treat infections by biofilm-forming bacteria are currently under development.

In this context, *H. pylori* is able to survive in an overly acidic gastric environment given the dual enzymatic system composed of Carbonic Anhydrases (CAs) and urease. Their complex interplay acts as a buffering system to maintain an almost neutral cell pH, ensuring the survival of the bacterium since it could not tolerate the acid pH (about 2.0) of the stomach mucus layer [16,17,18]. CAs are a superfamily of ubiquitary metalloenzymes, mainly containing Zn^2+^, involved in the reversible hydration of carbon dioxide to give a bicarbonate ion and a proton. Besides humans, these enzymes are also found in bacteria and, in particular, in *H. pylori*. Recently, two isoforms belonging to the α- and the β- classes, namely *Hp*αCA (*H. pylori*αCA) and *Hp*βCA (*H. pylori*βCA) [19,20,21], were characterized and the development of their inhibitors as antibacterial agents was reported [22,23,24,25,26,27,28]. Moreover, we previously demonstrated the presence of the α isoform of *H. pylori* CA in the outer membrane vesicles (OMVs), spherical lipidic structures released by the bacterium during its growth, in both planktonic (pOMVs) and biofilm (bOMVs) phenotypes. In particular, we demonstrated that the α-CA is majorly expressed in pOMVs compared to bOMVs and its activity increased over time in pOMVs. These data lead us to hypothesize a new role of this enzyme concerning the colonization and survival of *H. pylori* [21,29].

In the continuous race to discover biological-activities-endowed natural compounds, we focused our attention on a small molecule, carvacrol (CAR), a monoterpene phenol, abundant in several medicinal plants. This compound displays antimicrobial, food-preserving, antioxidant and anticancer activities [30]. In a previous work, we highlighted the ability of CAR to prevent *H. pylori* biofilm formation by inhibiting its CAs without affecting the viability of probiotic bacterial strains, i.e., *Limosilactobacillus reuteri* DSM 17938, *Lactobacillus rhamnosus* GG ATCC 53,103 and *Lactobacillus acidophilus* ATCC SD5214 [31]. Indeed, due to its mechanism of action, CAR is able to destabilize *H. pylori* OMV production, inducing a reduction in cell adhesion and aggregation [31]. In addition, Sisto and colleagues synthesized a series of CAR derivatives endowed with notable anti-*H. pylori* activity that showing improved Minimal Inhibitory Concentration (MIC) values compared to the parent compound CAR [32].

On the other hand, the inhibition of *H. pylori* urease could represent a new mechanism by which bacterial growth can be counteracted. As proof, several studies showed that, besides bacterial CAs, urease inhibition can also impair bacterial growth and reduce the expression of virulence factors [24,33,34]. Urease is a nickel-containing enzyme encoded by several microorganisms and plants since it catalyzes the hydrolysis of urea into ammonia and carbamate, which spontaneously decomposes into ammonia and bicarbonate ions, representing an essential player in their survival and/or pathogenicity. In *H. pylori*, the ureolytic activity allows the colonization of the host stomach, favoring the progression of the infection and, thus, gastritis, peptic ulcers and gastric cancers. Furthermore, *H. pylori* urease can cause a stimulation of the host inflammatory reaction and damage to tight junctions, resulting in a serious cytotoxic effect on the host, i.e., the increase in blood platelet aggregation in gastritis and cardiovascular diseases [35].

Among hydroxamic acids, well-known as urease inhibitors, Acetohydroxamic acid (AHA) (Lithostat^®^), already approved by the Food and Drug Administration (FDA) to be used in patients with chronic urea-splitting urinary infection, have shown a significant bacteriostatic effect against *H. pylori* species [33]. AHA is one of the most-studied compounds as potential therapeutics for the treatment of ulcers caused by *H. pylori* infection. In particular, AHA acts by chelating Ni^+^ ions in the active site and can be used synergistically with colloidal bismuth subcitrate, tetracycline, metronidazole and AMX [36]. However, some limitations due to serious side effects—such as psycho-neurological, musculo-integumentary symptoms and teratogenicity—were established for this therapy [37]. Another member of the hydroxamate-based urease inhibitor class is Salicylhydroxamic acid (SHA), also endowed with anti-inflammatory effects and currently used in clinics for both urinary and protozoa infections [38], with fewer side effects than AHA. Among the multiple biological activities, the SHA prevents the formation of phosphate stones by inhibiting urease activity. The SHA has a potent antispasmodic action on urethras, preventing renal colic, and has an analgesic effect 1.65 times more potent than acetylsalicylic acid. Recent research has demonstrated the *H. pylori* bactericidal activity of new bismuth(III) hydroxamate complexes derived from some hydroxamic acids, such as SHA [34]. Based on these observations, the multiple association of an inhibitor of both *Hp*CAs, a urease inhibitor and a current antibiotic, i.e., AMX, might represent a new strategy to fight *H. pylori* infection by acting on different essential bacterial enzymes.

In particular, we put our efforts into the study of CAR, SHA and AMX association to find their perfect combination to exert the highest antimicrobial and antibiofilm activity against *H. pylori* ATCC 43504. The choice to employ AMX as a reference antibiotic in this study is due to its use in first-line treatment against *H. pylori* infections in countries where clarithromycin resistance is >15% [8].

## 2. Results

### 2.1. MIC and MBC Determination of Carvacrol, Amoxicillin and Salicylhydroxamic Acid, Alone and in Combination with the Checkerboard Method

A checkerboard assay was performed to verify a possible synergistic effect between CAR, SHA and AMX, tested two by two, and Minimal Inhibitory Concentration (MIC) and Minimal Bactericidal Concentration (MBC) values of the drugs alone and in combination were measured. The results demonstrated that the best combinations were CAR-AMX and CAR-SHA, as confirmed by the Fractional Inhibitory Concentration index (FICI) of 0.75 which defined the combinations as additive (Table 1). The last combination, SHA-AMX, was less effective, as demonstrated by the FICI of 1.25 which was indifferent (Table 1).

### 2.2. Time Killing Assay

**Figure 1:**
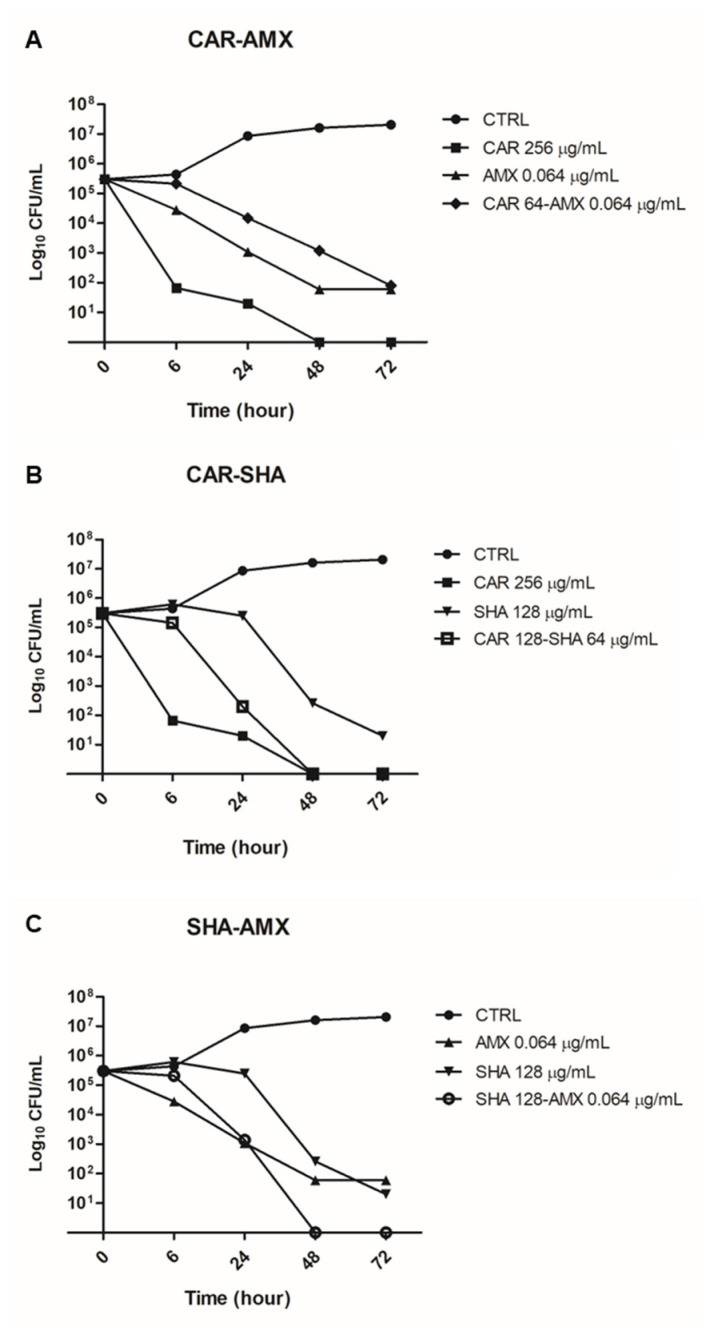
Bactericidal curves of the three compounds and their combinations vs. *H. pylori* ATCC 43504. (**A**) CAR and AMX alone and in association; (**B**) CAR and SHA alone and in association; (**C**) SHA and AMX alone and in association.

Killing curves were carried out to evaluate the rate of killing of *H. pylori* ATCC 43504 by CAR, AMX and SHA tested alone and in the combinations CAR-AMX, CAR-SHA and SHA-AMX at different time points (6, 24, 48 and 72 h). In the treatment of *H. pylori* with the CAR-AMX combination, AMX lowered the bactericidal concentration of CAR from 256 to 64 µg/mL after 72 h of incubation, as shown in Figure 1A.

The combination of CAR and SHA proved to be effective in reducing the bactericidal concentration of both molecules (Figure 1B). CAR and SHA alone had an MBC value of 256 and 128 µg/mL after 24 and 48 h of incubation, respectively. The CAR-SHA association decreased the MBC value by half after 48 h of incubation; these data underlined their synergy in *H. pylori* killing. The combination of SHA and AMX was also the least effective concerning bactericidal activity, beyond the MIC value. SHA-AMX bactericidal activity was detected after 48 h of incubation, at both the same concentration and the time of the individually tested molecules (Figure 1C).

### 2.3. MBEC Determination of Carvacrol, Amoxicillin and Salicylhydroxamic Acid, Alone and in Combination vs. H. pylori

**Figure 2:**
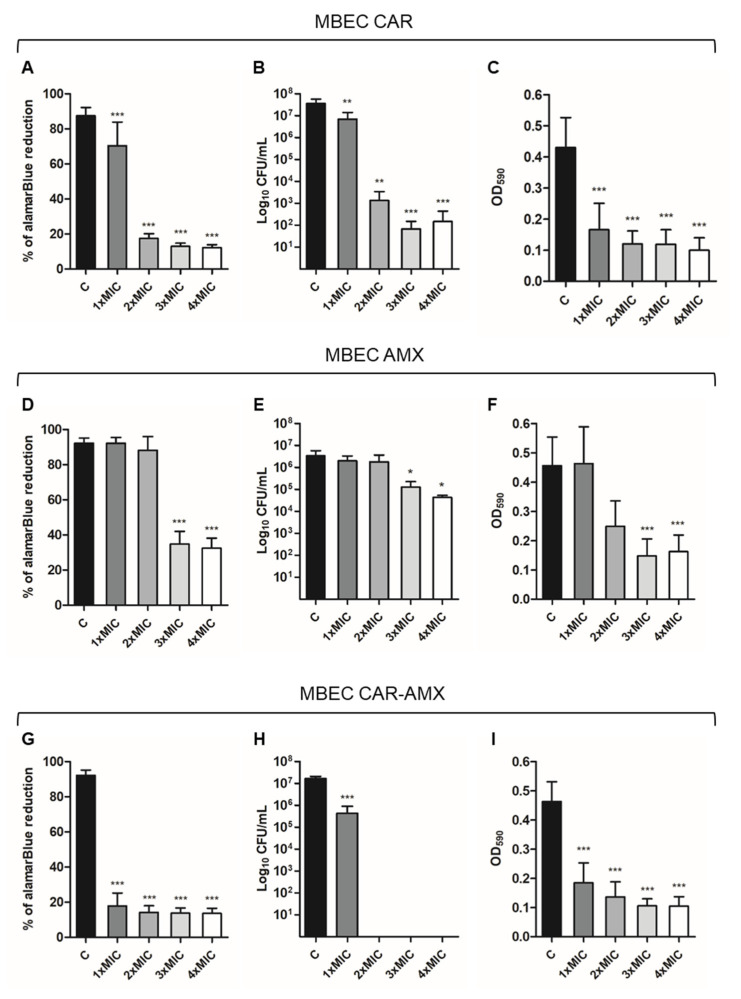
In vitro activity of CAR and AMX alone and in combination against *H. pylori* preformed biofilm. C: control biofilm (Brucella Broth with the addition of DMSO and H_2_O at the concentrations present in 1×, 2×, 3×, 4×MIC); 1×MIC: biofilm treated with CAR and AMX at MIC concentrations (CAR 64 µg/mL-AMX 0.008 µg/mL); 2×MIC: biofilm treated with CAR and AMX at 2×MIC concentrations (CAR 128 µg/mL-AMX 0.016 µg/mL); 3×MIC: biofilm treated with CAR and AMX at 3×MIC concentrations (CAR 192 µg/mL-AMX 0.024 µg/mL); 4×MIC: biofilm treated with CAR and AMX at 4×MIC concentrations (CAR 256 µg/mL-AMX 0.032 µg/mL). Histograms represent the MBEC of (**A**–**C**) CAR alone; (**D**–**F**) AMX alone; (**G**–**I**) CAR and AMX in association via (**A**,**D**,**G**) alamarBlue assay, (**B**,**E**,**H**) the CFU counting, and (**C**,**F**,**I**) the crystal violet assay, respectively. The results represent the mean ± SD of three independent experiments. * *p* < 0.05 vs. C, ** *p* < 0.005 vs. C; *** *p* < 0.001 vs. C (ANOVA + Dunnett’s multiple comparisons test).

Antibiofilm activity was evaluated by the determination of the Minimum Biofilm Eradication Concentration (MBEC). A preformed *H. pylori* biofilm, developed after 2 days of incubation, was treated with double MIC concentrations of CAR, AMX and SHA alone and in the following combinations: CAR-AMX, CAR-SHA and SHA-AMX. The CAR-AMX association resulted in a reduction of the MIC values of individually tested AMX and CAR. In the present study, we compared different concentrations of the two compounds against *H. pylori* biofilm. We assessed the MIC value of the CAR-AMX association by testing it at two different compound concentrations: the first by considering their individual MICs (Figure S1A–F) and the second at the concentrations of CAR and AMX used in association (Figure 2A–F). In particular:

- CAR was used at the concentration corresponding to the MIC in association with AMX (64 µg/mL) and 2×, 3× and 4× that value (64 µg/mL, thus: 128, 192, 256 µg/mL, respectively) (Figure 2A–C). In addition, it was used at the concentration corresponding to its own MIC (128 µg/mL, MIC of CAR alone) and to 2×, 3× and 4× that value (128 µg/mL, thus: 256, 384, 512 µg/mL, respectively) (Figure S1A–C).

- AMX was used at the concentration corresponding to the MIC in association with CAR (0.008 µg/mL) and to 2×, 3× and 4× that value (0.008 µg/mL, thus: 0.016, 0.024, and 0.032 µg/mL, respectively) (Figure 2D–F). Moreover, it was used at the concentration corresponding to its own MIC (0.032 µg/mL, MIC of AMX alone) and to 2×, 3× and 4× that value (0.032 µg/mL, thus: 0.064, 0.096, and 0.128 µg/mL, respectively) (Figure S1D–F).

**Figure 3:**
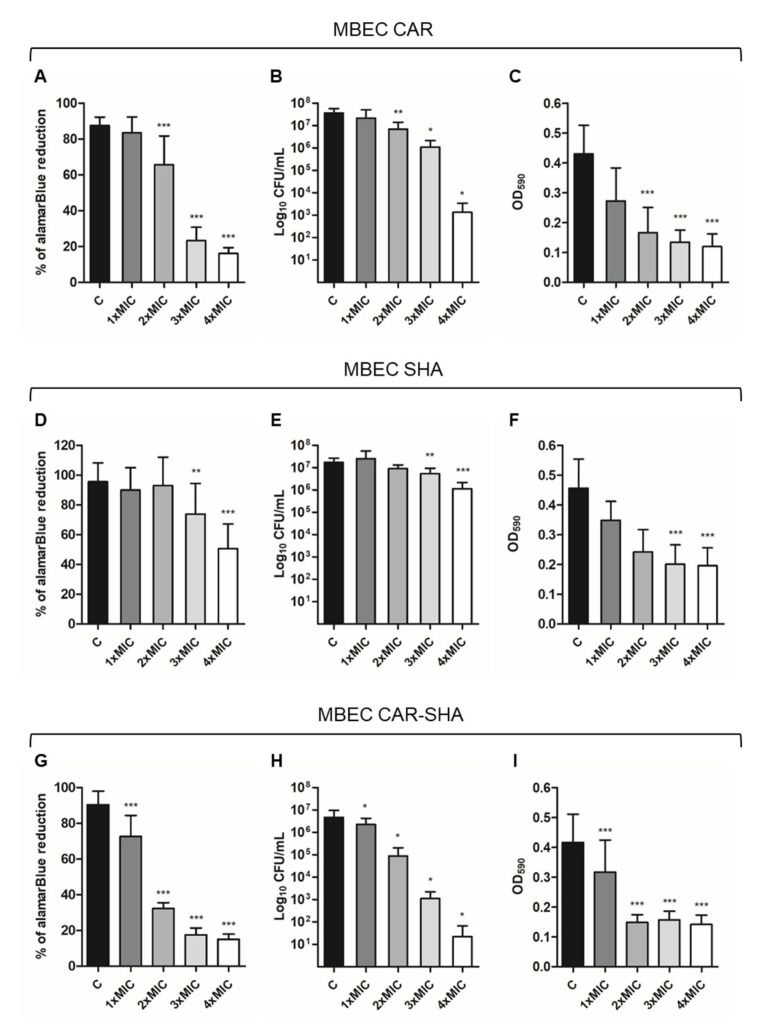
In vitro activity of CAR and SHA alone and in combination against *H. pylori* preformed biofilm. C: control biofilm (Brucella Broth with the addition of DMSO and ethanol/H_2_O at the concentrations present in 1×, 2×, 3×, 4×MIC); 1×MIC: biofilm treated with SHA and CAR at MIC concentrations (32 µg/mL); 2×MIC: biofilm treated with SHA and CAR at 2×MIC concentrations (64 µg/mL); 3×MIC: biofilm treated with SHA and CAR at 3×MIC concentrations (96 µg/mL); 4×MIC: biofilm treated with SHA and CAR at 4×MIC concentrations (128 µg/mL). Histograms represent the MBEC of (**A**–**C**) CAR alone; (**D**–**F**) SHA alone; (**G**–**I**) CAR and SHA in association via (**A**,**D**,**G**) alamarBlue assay, (**B**,**E**,**H**) the CFU counting, and (**C**,**F**,**I**) the crystal violet assay, respectively. The results represent the mean ± SD of three independent experiments. * *p* < 0.05 vs. C, ** *p* < 0.005 vs. C; *** *p* < 0.001 vs. C (ANOVA + Dunnett’s multiple comparisons test).

**Figure 4:**
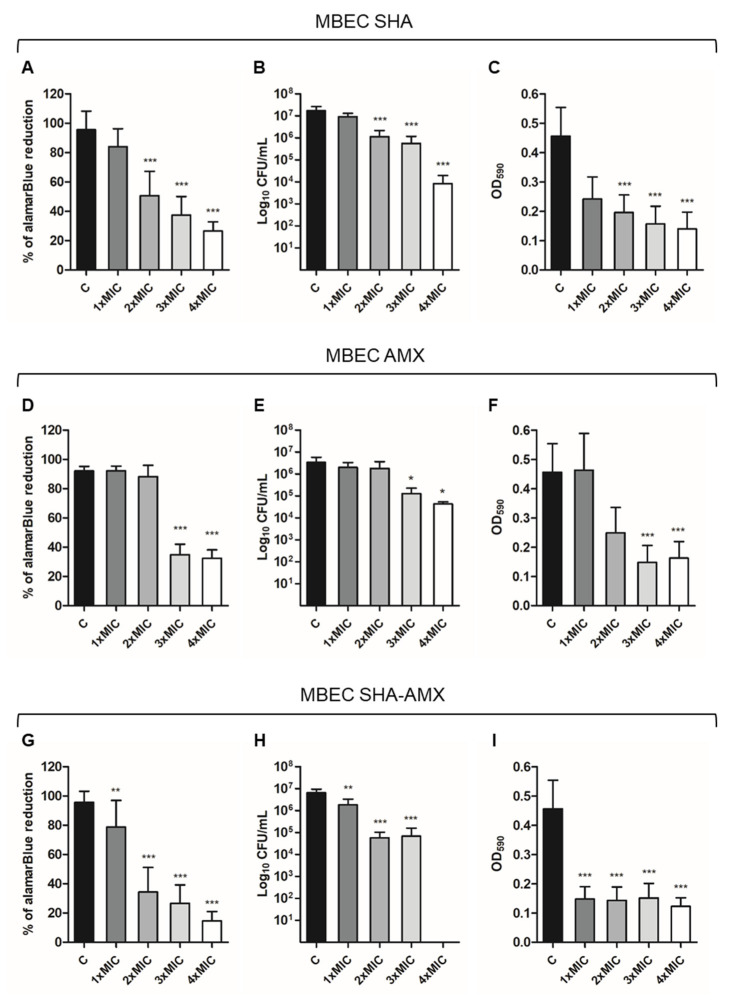
In vitro activity of SHA and AMX alone and in combination against *H. pylori* preformed biofilm. C: control biofilm (Brucella Broth with the addition of ethanol and H_2_O at the concentrations present in 1×, 2×, 3×, 4×MIC); 1×MIC: biofilm treated with SHA and AMX at MIC concentrations (SHA 64 µg/mL-AMX 0.008 µg/mL); 2×MIC: biofilm treated with SHA and AMX at 2×MIC concentrations (SHA 128 µg/mL-AMX 0.016 µg/mL); 3×MIC: biofilm treated with SHA and AMX at 3×MIC concentrations (SHA 192 µg/mL-AMX 0.024 µg/mL); 4×MIC: biofilm treated with SHA and AMX at 4×MIC concentrations (SHA 256 µg/mL-AMX 0.032 µg/mL). Histograms represent the MBEC of (**A**–**C**) SHA alone; (**D**–**F**) AMX alone; (**G**–**I**) SHA and AMX in association via (**A**,**D**,**G**) alamarBlue assay, (**B**,**E**,**H**) the CFU counting, and (**C**,**F**,**I**) the crystal violet assay, respectively. The results represent the mean ± SD of three independent experiments. * *p* < 0.05 vs. C, ** *p* < 0.005 vs. C; *** *p* < 0.001 vs. C (ANOVA + Dunnett’s multiple comparisons test).

As regards SHA, the MIC of individually tested SHA corresponded to the MIC of SHA in combination. The capability of the chosen combinations to eradicate *H. pylori* biofilm was determined by using alamarBlue (AB) assay, Colony Forming Unit (CFU) counting and crystal violet assay and was further compared to the treatment of the compounds that were used alone (Figure 2, Figure 3, Figure 4 and Figure S1).

#### 2.3.1. Combination of Carvacrol and Amoxicillin (CAR-AMX)

The results obtained showed that the treatments of *H. pylori* biofilm with CAR and AMX used alone at the MIC values in association (CAR 64 µg/mL and AMX 0.008 µg/mL) were less effective when compared to the effect resulting from their association (Figure 2). In particular, CAR was capable of reducing, but not completely eradicating, *H. pylori* biofilm at a concentration 1×MIC as demonstrated by all the three methods used (Figure 2A–C). Conversely, AMX used alone was effective at a concentration corresponding to 3×MIC (Figure 2D–F). Interestingly, the association of CAR-AMX induced a significant removal of the biofilm (Figure 2G–I). Of note, the CAR-AMX combination showed a synergy in reducing the biofilm biomass already at 1×MIC concentration with complete eradication of the biofilm at 2×MIC, as shown by the total absence of CFU count in Figure 2H and a statistically significant reduction of the percentage of alamarBlue and the crystal violet absorbance (Figure 2G,I).

We also determined the MBEC of individually tested CAR and AMX vs. *H. pylori* at concentrations equal to their own MICs and in the range of 1–4×MIC. The results showed that CAR and AMX used alone were effective in eradicating *H. pylori* biofilm at high concentrations, corresponding to 128 µg/mL and 0.032 µg/mL, respectively (Figure S1). On the contrary, the concentrations used in the association were 64 µg/mL and 0.008 µg/mL, respectively (Figure 2). The obtained results further validated the synergistic effect of CAR-AMX vs. *H. pylori* biofilm.

#### 2.3.2. Combination of Carvacrol and Salicylhydroxamic Acid (CAR-SHA)

The results of biofilm eradication by CAR and SHA alone and in combination at different concentrations are presented in Figure 3. The CAR-SHA association reduces *H. pylori* biofilm at 2×MIC concentration with total eradication of the biofilm at 4×MIC. The CAR-SHA combination provokes a 50% reduction in both alamarBlue and crystal violet absorbance at 2×MIC concentration with a statistically significant reduction of 2Log_10_CFU/mL (Figure 3G–I). The effective combination of CAR-SHA in reducing the biofilm was validated by the results obtained by biofilm treatment with CAR and SHA alone (Figure 3A–F). The treatments with CAR and SHA alone are less effective in reducing *H. pylori* biofilm, as shown by all three assays.

#### 2.3.3. Combination of Salicylhydroxamic Acid and Amoxicillin (SHA-AMX)

The treatments with SHA and AMX alone were less effective in reducing *H. pylori* biofilm, as shown by all three assays (Figure 4A–F). In particular, a significant reduction in CFU counts was detectable at 2×MIC and 3×MIC concentrations, respectively (Figure 4B,E). The combination of SHA-AMX in reducing the biofilm was validated by the results obtained by the biofilm treatment with SHA and AMX alone (Figure 4A–F). The SHA-AMX combination showed a synergy in reducing the biofilm biomass at 2×MIC concentration, where the percentage of reduction relating to alamarBlue was below 50% and a statistically significant decrease of 2Log_10_CFU/mL was detected (Figure 4G,H). Despite being effective in eradicating *H. pylori* biofilm, SHA-AMX is less powerful compared to the CAR-AMX combination.

### 2.4. MBIC of Carvacrol, Amoxicillin and Salicylhydroxamic Acid, Alone and in Combination, vs. H. pylori ATCC 43504/NCTC11637

The antibiofilm activities of CAR, SHA and AMX alone and in combination were determined by the Minimum Biofilm Inhibitory Concentration (MBIC) assay. The sub-MIC doses of CAR-AMX, CAR-SHA and AMX-SHA did not show efficacy in inhibiting *H. pylori* biofilm formation compared both to the control and the compounds tested alone (Supplementary Materials).

### 2.5. Effects of Carvacrol, Amoxicillin and Salicylhydroxamic Acid, Alone and in Combination, on H. pylori Ultrastructure

**Figure 5:**
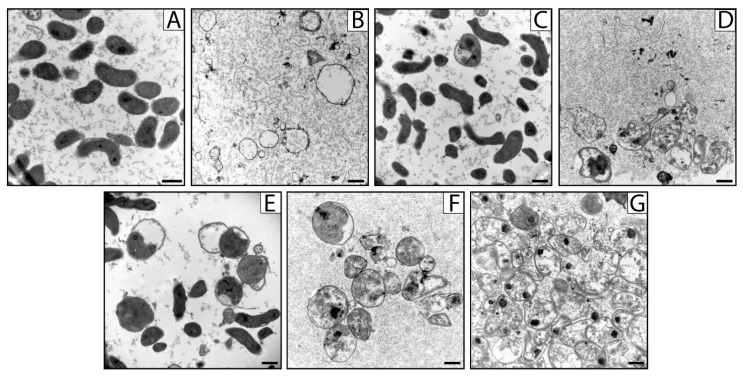
TEM representative images of *H. pylori* cells untreated (**A**) at 72 h of incubation with CAR (**B**), SHA (**C**) and AMX (**D**) as well as with the combinations CAR-AMX (**E**), SHA-AMX (**F**) and CAR-SHA (**G**) at MIC concentrations. Scale bars: 500 nm.

The effects on the ultrastructure of *H. pylori* cells of CAR, AMX and SHA alone and their combinations corresponding to CAR-AMX, SHA-AMX and CAR-SHA were examined by using Transmission Electron Microscopy (TEM) analysis at MIC concentrations after 72 h of incubation (Figure 5). Controls, corresponding to untreated bacteria, possessed the typical spiral/rod-shaped morphology of *H. pylori*. Some of them showed a coccoid phenotype, characteristic of 72 h of incubation: the cell wall was intact and enclosed a dense cytoplasm (Figure 5A). *H. pylori* cells treated with CAR alone (128 µg/mL) displayed a prevalent coccoid morphotype characterized by membrane structures surrounding apparently empty cells—commonly known as ‘ghosts’ corresponding to empty bacterial cell envelopes without cytoplasm and DNA (Figure 5B). The treatment with SHA alone (64 µg/mL) led to occasional swelling of the periplasmic space, but the bacteria maintained their integrity and structure (Figure 5C). Figure 5D showed *H. pylori* treated with amoxicillin at MIC concentrations (0.032 µg/mL). Broken cell membranes with the release of cytosolic material outside were visible.

The treatment with CAR and AMX (CAR two-fold decrease and AMX four-fold decrease compared to earlier-described samples) led to more frequent swelling of the periplasmic spaces. The cells also appeared swollen, leading to a reduced intensity of cytoplasmic staining (Figure 5E). The treatment of the samples with AMX and SHA (AMX eight-fold decrease than earlier described samples while SHA unaltered) led to the swelling of cells and very efficient permeabilization resulting in membrane ghosts and sparsely stained cytoplasm (Figure 5F). Finally, the treatment of cells with CAR and SHA (CAR four-fold decrease and SHA two-fold decrease compared to earlier-described samples) resulted again in an efficient permeabilization and swelling of the cells resulting in sparse cytoplasm. In addition, in this condition, the membranes were also frequently lysed, and the spherical morphology of the cell was not maintained (Figure 5G).

## 3. Discussion

The therapies used according to the current consensus guidelines often fail to achieve the eradication of *H. pylori* due to the high rate of antimicrobial resistance and the difficulty for antimicrobials to cross the EPS matrix of *H. pylori* biofilm [11,39,40,41,42,43]. For this reason, there is an urgency to discover the compounds targeting bacterial biofilms and/or bacterial enzymes involved in essential pathways, with a different mechanism of action compared to the common antimicrobials. Among them, carbonic anhydrases and urease emerged as new valuable pharmacological targets for the development of innovative antimicrobial drugs. Besides AMX, one of the most employed antibiotics in clinics, CAR and SHA are becoming a great focus of interest in this field since recent studies demonstrated their effectiveness vs. drug-sensitive and resistant *H. pylori* strains [31,32,34]. In this context, the antimicrobial and antibiofilm activities of CAR, AMX and SHA alone, as well as their combinations corresponding to CAR-AMX, CAR-SHA and SHA-AMX, against *H. pylori* were investigated.

The data obtained in the current study showed greater efficacy for antimicrobial and antibiofilm activity in vitro of the combinations CAR-AMX, CAR-SHA and SHA-AMX vs. *H. pylori* compared to the same compounds used alone. CAR-AMX was the best combination vs. *H. pylori* and was able to reduce both the MIC and the MBC values compared to the individually tested molecules. These data represent an important result considering that *H. pylori* is the aetiologic agent of gastric cancer. As regards the biofilm eradication, the CAR-AMX association reduces the biofilm biomass at 2×MIC concentration, a lower concentration with respect to the concentrations of the molecules used alone at the concentrations which were active in combination.

We previously demonstrated that *H. pylori* biofilm matrix contains OMVs associated with extracellular DNA (eDNA). Such structures contribute to the stabilization of the biofilm via the interaction between OMVs and between OMVs and cells [29]. In addition, we showed that CAR, a well-characterized and selective *Hp*CA inhibitor, is capable of inhibiting the production of OMVs in *H. pylori*, thus preventing biofilm formation [31]. On the contrary, amoxicillin did not prevent the release of OMVs, as shown by the similar results of the AMX-treated samples and the controls. Moreover, a large percentage of OMVs released delivered eDNA, probably contributing to the horizontal gene transfer mechanism [31]. Carvacrol was able to inhibit biofilm formation associated with the release of OMVs at a concentration of 64 µg/mL. Conversely, in the present study, we tested a lower concentration of CAR corresponding to 32 µg/mL, the same used in association with AMX. In this case, CAR did not inhibit biofilm development. We speculate that the capability of carvacrol to eradicate *H. pylori* biofilm is probably due to its ability to permeabilize or destroy OMVs, creating a passage via the EPS biofilm matrix and facilitating the penetration of amoxicillin that reaches the target cell, inducing its death.

The overall study of the association of CAR-AMX might represent an important milestone since it involves the use of the two compounds active at lower concentrations, thus less toxic, as promising antibacterial agents for the treatment of *H. pylori* infections with a clinical purpose. The SHA showed good efficacy in reducing *H. pylori* planktonic growth, but a low potency in eradicating its biofilm; the killing curves show no synergy of SHA and AMX in reducing the MBC values. Recent studies have demonstrated that the SHA exhibited antibacterial activity against *H. pylori* with a MIC value > 25 µM [34], in accordance with our experiments. The authors demonstrated that new bismuth(III) hydroxamate complexes, derived from hydroxamic acids, showed greater antimicrobial activity against planktonic *H. pylori,* even if no investigation on the antibiofilm efficacy was provided. However, hydroxamic acids and their complexes were found to be non-toxic to human fibroblast cells, up to 100 μM [34].

It is well known that AMX is degraded by gastric acids; therefore, higher concentrations of the drug are necessary to reach a therapeutic effect against *H. pylori* infection, often resulting in a worsening of the gastrointestinal side effects [44,45]. The effective association of CAR-AMX and SHA-AMX, as demonstrated in the present study, could lead to lowering the doses of amoxicillin necessary to eradicate the pathogen, improving the patient’s compliance.

Lastly, the association of CAR and SHA decreased the active concentrations of the two compounds individually tested, improving the eradication of *H. pylori* biofilm. These results proved that the inhibition of two bacterial metabolic essential pathways could be successful to counteract *H. pylori* infection. CAR and SHA in combination could be used at lower concentrations, less toxic than the MIC concentration of the molecules used alone.

Although the mechanism of action of CAR against *H. pylori* cells remains unknown, its biological effect vs. other bacterial species is well established [46,47,48]. Ultee and co-authors investigated CAR antimicrobial activity against the food-borne pathogen *Bacillus cereus,* which is hypothesized to have a compound chemical structure and, in particular, the presence of a hydroxyl group and delocalized electron-composed system. The latter could be responsible for its proton-exchanger activity, thereby reducing the pH gradient across the bacterial cytoplasmic membrane. The resulting collapse of the proton driving force and the depletion of the ATP pool would eventually lead to cell death [48]. In Gram-negative bacteria, carvacrol induces marked changes in both cell morphology and membrane permeability with loss of cytosolic material, as demonstrated by Ciandrini and co-authors [46]. TEM analysis displays membrane-breaking in *Porphyromonas gingivalis* as well as surface blebbing in both *P. gingivalis* and *Fusobacterium nucleatum* [46].

This study highlights, for the first time, the morphological changes of *H. pylori* cells induced by the treatment with carvacrol. At MIC concentration, carvacrol induced the formation of “ghosts”, as already observed in *Escherichia coli* O157:H7 [47], letting us hypothesize that it may promote the permeabilization of the outer membrane, probably facilitating the penetration of amoxicillin into the cell without cell wall lysis when used in association with each other. Amoxicillin, for its part, induces the formation of spheroplast and cell lysis at MIC concentration, as previously demonstrated by Horii and co-authors [49]. AMX binds to Penicillin-Binding Proteins (PBPs) provoking the inhibition of the biosynthesis of peptidoglycan, thus promoting cell wall lysis. This process could be related to the detachment of the inner membrane from the outer membrane as demonstrated by Makobongo and co-authors [50]. Moreover, TEM images showed that the CAR-AMX association increased coccoid cellular morphology. The research of the ultrastructural effect of urease inhibitors towards *H. pylori* has not been investigated in the literature. Sharaf and co-authors demonstrated that the bioflavonoid hesperetin-7-rhamnoglucoside inhibited *H. pylori* urease in a concentration-dependent manner [51]. In detail, bioimaging studies illustrated that this bioflavonoid interacted with *H. pylori* cells and induced membrane rupture by creating holes in the outer membrane of the bacterial cells, morphological changes as well as shrinkage of some bacterial cells [51]. On the other side, the urease inhibitor SHA, used in this study, did not provoke cellular lysis, underlining the fact that the *H. pylori* inhibition mechanism did not affect the integrity of the membrane integrity, different from what we observed in the SHA combination with AMX or CAR. The association SHA-AMX could target cytoplasmic content to induce the formation of electron-dense cytoplasmatic structures as well as the separation of the inner membrane from the outer membrane. The CAR-SHA combination demonstrated again that *H. pylori* essential enzyme inhibition provoked destruction of the cells, with ghosts and electron-dense cytoplasmic structures.

In conclusion, we developed and analyzed in-depth three associations of the active compounds CAR, SHA and AMX and their biological activities. Thus, they represent an innovative and promising therapeutical strategy against *H. pylori* infections since they contain low concentrations of AMX and target different bacterial enzymes. In particular, the decrease in AMX doses can correspond to a consequent reduction of the annoying gastrointestinal side effects and the development of AMX-resistant phenotypes. In addition, agents with new mechanisms of action, such as those involving the inhibition of *Hp*CAs and urease, might contribute to limiting both the spread of antimicrobial resistance and *H. pylori* pathogenicity. However, further investigations on the pharmacokinetics, pharmacodynamics and toxicology, as well as studies on in vivo animal models of the associated compounds, will be needed for a possible future application in the medical field to counteract *H. pylori* infections.

## 4. Materials and Methods

### 4.1. Bacterial Strain and Media

The reference strain *H. pylori* ATCC 43504, a well-characterized strain used in our previous study, was used in this study and was stored at −80 °C before being thawed at room temperature, plated on Columbia agar base (CA; Oxoid Limited, Basingstoke, Hampshire, UK) supplemented with 10% horse serum (Sigma Aldrich, St. Louis, MO, USA) and 0.25% bacto yeast extract (Oxoid Ltd., Basingstoke, Hampshire, UK) and finally incubated at 37 °C for 72 h in a microaerophilic atmosphere (Campygen; Oxoid Ltd., Basingstoke, Hampshire, UK) [52].

### 4.2. Antimicrobial Activity of Carvacrol, Amoxicillin and Salicylhydroxamic Acid vs. H. pylori ATCC 43504

The combination effect of CAR-AMX, CAR-SHA and SHA-AMX was determined by a checkerboard assay and evaluated by using the Fractional Inhibitory Concentration (FIC) index. The FIC index (FICI) was calculated from the Fractional Inhibitory Concentration (FIC) values of the test compounds and antimicrobials, by using the following formula:

(1) FICI = FIC_A_ + FIC_B_;
(2) FIC_A_ = (MIC_A_^comb^/MIC_A_^alone^) and FIC_B_ = (MIC_B_^comb^/MIC_B_^alone^), where MIC_A_^comb^ and MIC_B_^comb^ are the MIC values of the drugs A and B, respectively, in the combination AB and MIC_A_^alone^ and MIC_B_^alone^ are the MIC values of the drugs A and B individually evaluated;
(3) FBCI = FBC_A_ + FBC_B_;
(4) FBCI = (MBC_A_^comb^/MBC_A_^alone^) + (MBC_B_^comb^/MBC_B_^alone^), where MBC_A_^comb^ and MBC_B_^comb^ are the MBC values of the drugs A and B, respectively, in the combination AB and MBC_A_^alone^ and MBC_B_^alone^ are the MBC values of the drugs A and B individually evaluated.

The FICI/FBCI values ≤ 0.5, >4.0, and >0.5–4 were defined as synergistic, antagonist and non-synergistic or additive, respectively [52,53]. In detail, when FICI/FBCI is >0.5 to 1 the combination is additive; when FICI/FBCI is >1 to 4 the combination is indifferent.

The MIC was determined by the broth micro-dilution method, in 96-well microtiter plates, following the guidelines of the Clinical and Laboratory Standards Institute [54]. After 72 h of incubation, *H. pylori* colonies were collected and inoculated in a liquid culture medium consisting of Brucella Broth (BB; Oxoid Ltd., Basingstoke, Hampshire, UK,) supplemented with 2% (*v/v*) of fetal bovine serum (FBS; Sigma Aldrich). *H. pylori* broth cultures were subsequently incubated overnight (ON) at 37 °C under microaerophilic conditions, with shaking at 125 rpm. After 18 h of incubation, the broth cultures were diluted until reaching an OD_600_ of 0.20 (Spark^®^ multimode microplate reader, Tecan Trading AG, Mannedorf, Switzerland), corresponding to 2–8 × 10^7^ colony forming units/mL (CFU/mL) and further diluted to obtain 2–8 × 10^5^ CFU/mL per condition in the well. The compounds were then prepared for the MIC assay. The CAR was dissolved in DMSO; AMX was dissolved in type I water and the SHA was dissolved in hydroalcoholic solution (50% *v/v* water/ethanol). The three molecules were then diluted in *H. pylori* liquid culture medium described above. Serial dilutions of CAR, SHA and AMX were carried out to obtain the concentration ranges of 32–512 µg/mL, 16–256 µg/mL and 0.008–0.128 µg/mL, respectively. The residual percentage of DMSO was 0.75%; the residual percentage of water/ethanol was 2.31% for each one. Therefore, the growth of *H. pylori* in the presence of 0.75% of DMSO and 2.31% of water/ethanol was also evaluated. The plates were then incubated at 37 °C for 72 h under microaerophilic conditions. After 72 h of incubation, the MIC was defined via the alamarBlue assay (AB; Thermo Fisher Scientific, Waltham, MA, USA), as previously described [31]. AlamarBlue was added at 10% in each well, following the manufacturer’s instructions, and the plates were incubated for 4 h at 37 °C in a microaerophilic atmosphere. After the incubation, the percentage of reduction relating to alamarBlue was calculated, as previously described [55]. Afterwards, the MBC was determined, starting from the wells stained with alamarBlue: 100 μL of the bacterial suspensions into the blue-violet wells were seeded on the selected agar plates and incubated at 37 °C for 5 days in microaerophilic conditions. The MBC was defined as the lowest concentration of the tested combinations capable of killing the initial bacterial inoculum beyond 99.9%. Three independent experiments were performed in triplicate.

### 4.3. Time Killing Assay of CAR, AMX and SHA and their Combinations vs. H. pylori ATCC 43504

The bactericidal activity of CAR, AMX, SHA and their combinations was also evaluated by killing kinetics. *H. pylori* was cultivated in the liquid medium mentioned above and in the presence of the molecules at a concentration equal to the MBC. After 0, 6, 24, 48 and 72 h of incubation, the number of CFU was assessed by plating serial dilution of each sample onto the selected agar plates. The rate of killing was expressed as a viable count (log_10_ CFU/mL) against time. The control without the test molecules was also carried out.

### 4.4. Antibiofilm Activity of Carvacrol, Amoxicillin and Salicylhydroxamic Acid, Alone and in Combination, vs. H. pylori ATCC 43504/NCTC11637

The antibiofilm activity was evaluated by the determination of the MBIC and the MBEC. The MBIC was established by treating *H. pylori*-forming biofilm with sub-MIC concentrations of CAR, AMX and SHA, alone and in combination, following the previously published protocol [31]. CAR, AMX and SHA were dissolved as described above and diluted until the sub-MIC doses were obtained. The compounds alone were tested at the following concentrations: 0.004 µg/mL for AMX, 16 and 32 µg/mL for CAR, 16 and 32 µg/mL for SHA; The compounds in combination were tested at the following concentrations: 0.004 µg/mL for AMX in combination with 32 µg/mL of CAR, 0.004 µg/mL for AMX in combination with 32 µg/mL of SHA, 16 µg/mL for SHA in combination with 16 µg/mL of CAR. For each condition, *H. pylori* was inoculated at a concentration such that approximately 2–8 × 10^6^ CFU/mL are in the well. The antibiofilm assay was performed in 96-well flat bottom microtiter plates (Falcon, Corning, NY, USA). Three independent experiments were performed in triplicate.

Regarding the MBEC of CAR, AMX and SHA, the biofilms were developed as previously described [52]. Briefly, *H. pylori* biofilm was formed in the same medium used for the MBIC and starting from the same inoculum of 2–8 × 10^6^ CFU/mL. At the end of incubation, the biofilms were washed in Phosphate Buffered Saline (PBS; Sigma Aldrich, St. Louis, MO, USA) and the test compounds, alone and in combination, were added to the mature biofilms at concentrations corresponding to 1×MIC, 2×MIC, 3×MIC and 4×MIC. In every experiment, the growth control, consisting of *H. pylori* biofilm without the test compounds, was inserted.

After the incubation of MBIC and MBEC experiments, the antibiofilm activity was determined via the alamarBlue assay, the CFU count and the crystal violet assay (CV; Sigma Aldrich, St. Louis, MO, USA). In detail, at the end of the incubation, the non-adherent cells were removed, and the biofilms were washed with 100 µL of PBS. AlamarBlue was diluted 1:10 in Brucella Broth and 100 µL was added to each biofilm in the wells. The biofilms were incubated for 5 h at 37 °C in microaerophilic conditions. At the end of the incubation, the percentage of alamarBlue reduction was determined as described above. The CFU count was carried out by collecting bacteria from the wells stained with alamarBlue. Serial dilutions of *H. pylori*-treated and untreated biofilms were carried out in PBS, plated on the selected agar and incubated at 37 °C under microaerophilic conditions, for 5 days.

The crystal violet assay was used to determine the biofilm biomass. After PBS washing, the biofilms were dried for 1 h at 60 °C and stained with 0.5% crystal violet for five minutes at room temperature; the crystal violet was removed and the biofilms were washed with 100 µL of sterile water; then, the biofilms were dried for 30 min at room temperature and 100 µL acetic acid (Sigma-Aldrich)—diluted until 33% with sterile water—was added to the wells. After 10 min, the absorbance at 590 nm was measured.

### 4.5. TEM Analysis of H. pylori Treated with CAR, AMX, SHA and Their Combinations CAR-AMX, CAR-SHA and SHA-AMX

TEM analysis was carried out to evaluate the ultrastructure of *H. pylori* cells treated with CAR, AMX, SHA and the combinations corresponding to CAR-AMX, SHA-AMX and CAR-SHA at concentrations equal to the MIC. *H. pylori* broth cultures were prepared in the growing medium with the compounds mentioned above at MIC concentration (Table 1). Eight mL of each treated broth culture was incubated at 37 °C for 72 h in a microaerophilic atmosphere; then, eight mL of *H. pylori* in its growing medium were also prepared as a control. After the incubation, the broth cultures were centrifuged at 3200× *g* at 4 °C for 20 min, and the pellets were fixed by gently resuspending in fixing solution (2.5% glutaraldehyde +2% paraformaldehyde in 0.1 M sodium cacodylate buffer, pH 7.4) for 2 h at room temperature. After centrifugation, all pellets were rinsed in 0.1 M sodium cacodylate buffer (pH 7.4) and post-fixed in 1% osmium tetroxide in the same buffer for 1 h at room temperature. The pellets were first washed in bi-distilled water and stained with 1% uranyl acetate (30 min at room temperature in the dark), rinsed and dehydrated in a series of graded steps of ethanol (30, 50, 70, 95, 100%), two times with 100% of acetone, following embedding in epoxy resin and polymerization at 60 °C for 72 h. The polymerized blocks were trimmed using a Leica EM UC7 ultramicrotome (Leica Microsystems) and the ultrathin (70 nm) sections were collected and mounted on grids. Thin section images were acquired using an FEI Tecnai 12 BioTwin transmission electron microscope (FEI, Hillsboro, OR, USA) equipped with a Veleta CCD digital camera (Olympus Soft Imaging Solutions, Munster, Germany).

## Figures and Tables

**Table 1:** MIC and MBC values for CAR, AMX and SHA, alone and in combination, and their corresponding FICI and Fractional Bactericidal Concentrations Index (FBCI) against *H. pylori* ATCC 43504 through the checkerboard method.

*H. pylori* ATCC 43504	CAR [µg/mL]		AMX [µg/mL]		SHA [µg/mL]		FICI ^a^	FBCI ^b^
	MIC	MBC	MIC	MBC	MIC	MBC		
DRUG ALONE	128	256	0.032	0.064	64	128	-	-
COMBINATIONS								
CAR-AMX	64	64	0.008	0.064	-	-	0.75	1.25
CAR-SHA	32	64	-	-	32	64	0.75	0.75
SHA-AMX	-	-	0.008	0.064	64	128	1.25	2

^a^ FICI = (MIC_A_^comb^/MIC_A_^alone^) + (MIC_B_^comb^/MIC_B_^alone^); ^b^ FBCI = (MBC_A_^comb^/MBC_A_^alone^) + (MBC_B_^comb^/MBC_B_^alone^); ^a–b^ In combinations of A and B, the letters indicate the drugs in the order they appear.

## Data Availability

The data presented in this study are available in the main text and Supplementary Materials.

## Supplementary Materials

The following supporting information can be downloaded at: https://www.mdpi.com/article/10.3390/ijms24054455/s1.

## Abbreviations

CAs: carbonic anhydrases; CAR, carvacrol; AMX, amoxicillin; SHA, salicylhydroxamic acid; MIC, minimal inhibitory concentration; MBC, minimal bactericidal concentration; TEM, transmission electron microscopy; OMVs, outer membrane vesicles; FIC, fractional inhibitory concentration; FICI, fractional inhibitory concentration index; FBC, fractional bactericidal concentrations; FBCI, fractional bactericidal concentrations index.
