# Supplementary material for: Antimicrobial and Antibiofilm Activities of Carvacrol, Amoxicillin and Salicylhydroxamic Acid Alone and in Combination vs. Helicobacter pylori: Towards a New Multi-Targeted Therapy
Source: Int J Mol Sci. 2023 Feb 24;24(5):4455. doi: 10.3390/ijms24054455 (PMC10002413; doi:10.3390/ijms24054455)
Supplement: Supplementary file 1 [file ijms-24-04455-s001.zip › ijms-2180275-supplementary.pdf]

Antimicrobial and Antibiofilm Activities of Carvacrol, Amoxicillin and Salicylhydroxamic Acid Alone and in Combination *versus Helicobacter pylori:* Towards a new multi-targeted therapy

Valentina Puca[1], Gabriele Turacchio[2], Beatrice Marinacci[1,3], Claudiu T. Supuran[4], Clemente Capasso[5], Pamela Di Giovanni[1], Ilaria D'Agostino[1], Simone Carradori[1,†], Rossella Grande[1,†,*]

[1]Department of Pharmacy, "G. d'Annunzio" University of Chieti-Pescara, 66100 Chieti, Italy; valentina.puca@unich.it (V.P.); beatrice.marinacci@unich.it (B.M.); pamela.digiovanni@unich.it (P.D.G.); ilaria.dagostino1@unich.it (I.D.A.); simone.carradori@unich.it (S.C.); rossella.grande@unich.it (R.G.)

[2]Institute of Endocrinology and Experimental Oncology "Gaetano Salvatore", National Research Council, 80131 Naples, Italy; gabriele.turacchio@ieos.cnr.it (G.T.)

[3]Department of Innovative Technologies in Medicine & Dentistry, "G. d'Annunzio" University of Chieti-Pescara, 66100 Chieti, Italy

[4]Neurofarba Department, Section of Pharmaceutical and Nutraceutical Sciences, University of Florence, 50019 Sesto Fiorentino, Florence, Italy; claudiu.supuran@unifi.it (C.T.S.)

[5]Department of Biology, Agriculture and Food Sciences, National Research Council (CNR), Institute of Biosciences and Bioresources, 80131 Naples, Italy; clemente.capasso@ibbr.cnr.it (C.C.)

†These authors contributed equally

*Corresponding author

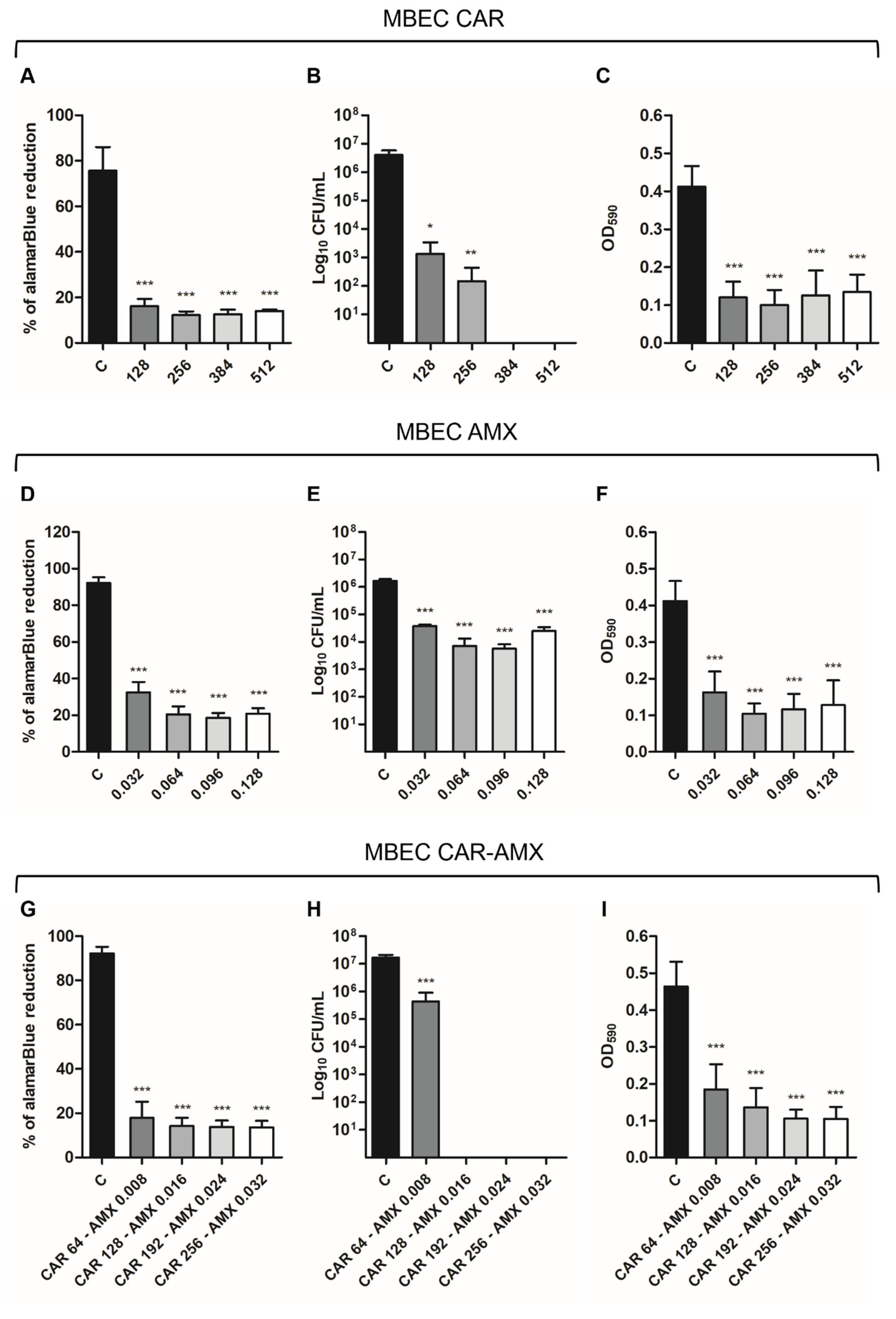
MBEC CAR
A
B
C
% of alamarBlue reduction
Log10 CFU/mL
OD590
C
128
256
384
512
MBEC AMX
D
E
F
C
0.032
0.064
0.096
0.128
MBEC CAR-AMX
G
H
I
C
CAR 64 - AMX 0.008
CAR 128 - AMX 0.016
CAR 192 - AMX 0.024
CAR 256 - AMX 0.032

Figure S1. *In vitro* eradicating activity (MBEC) of CAR and AMX alone (µg/mL) against *H. pylori* preformed biofilm. C: control biofilm; *H. pylori* biofilm was treated with: CAR and AMX at MIC concentrations (CAR 128 µg/mL and AMX 0.032 µg/mL); with CAR and AMX at 2xMIC concentrations (CAR 256 µg/mL and AMX 0.064 µg/mL); with CAR and AMX at 3xMIC concentrations (CAR 384 µg/mL and AMX 0.096 µg/mL) and with CAR and AMX at 4xMIC concentrations (CAR 512 µg/mL and AMX 0.128 µg/mL). (**A**–**C**) represent the MBEC of CAR alone; (**D**–**F**) represent the MBEC of AMX alone; (**G**–**I**) represent the MBEC of CAR and AMX in association *via* alamarBlue assay, the CFU count and the crystal violet assay, respectively. The results represent the mean ± SD of three independent experiments. * $p < 0.05$ *vs* C, ** $p < 0.005$ *vs* C; *** $p < 0.001$ *vs* C (ANOVA + Dunnett's Multiple comparison test).

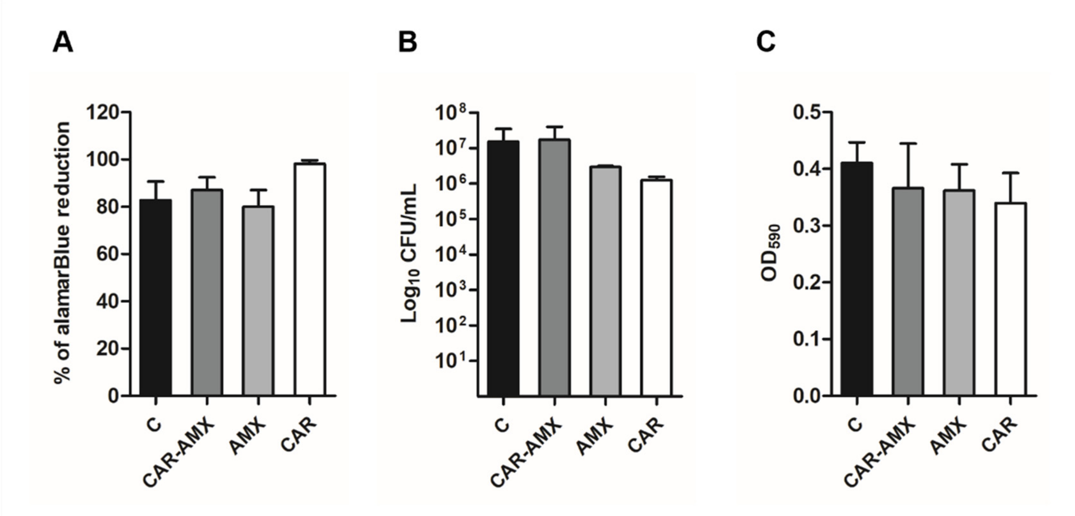

Figure S2. MBIC Determination of CAR and AMX, individually tested and in associations, *versus H. pylori via* alamarBlue assay (**A**), the CFU count (**B**) and the crystal violet assay (**C**). C: control biofilm.

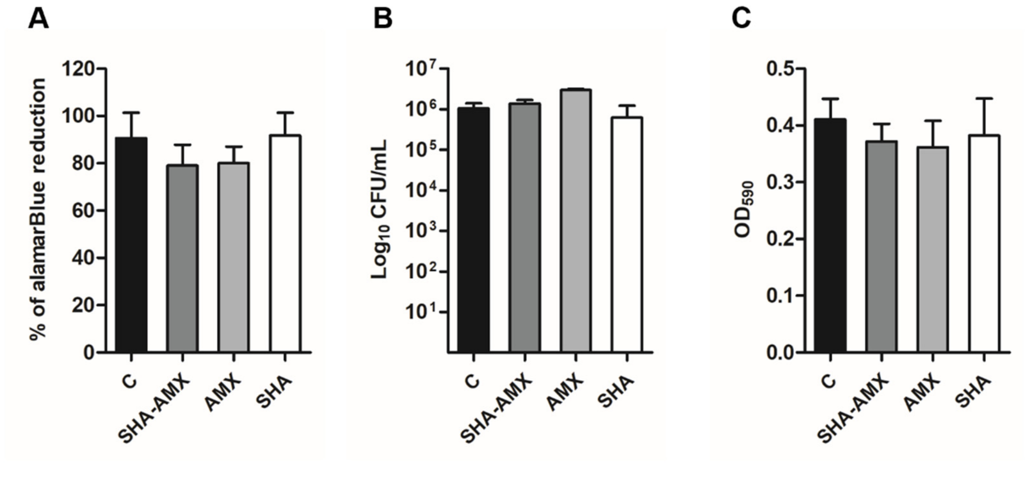

Figure S3. MBIC Determination of SHA and AMX, individually tested and in associations, *versus H. pylori via* alamarBlue assay (**A**), the CFU count (**B**) and the crystal violet assay (**C**). C: control biofilm.

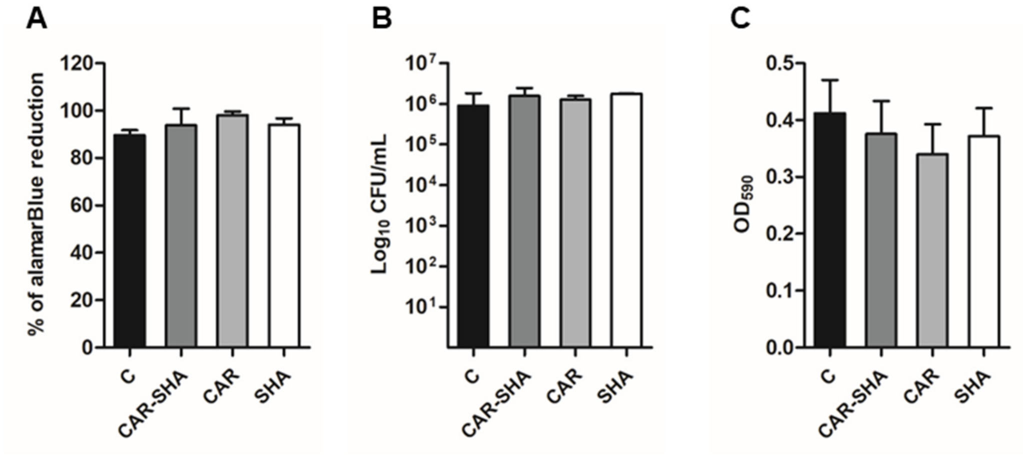

Figure S4. MBIC Determination of CAR and SHA, individually tested and in associations, *versus H. pylori via* alamarBlue assay (**A**), the CFU count (**B**) and the crystal violet assay (**C**). C: control biofilm.
